# Moderate sensitivity and high specificity of emergency department administrative data for transient ischemic attacks

**DOI:** 10.1186/s12913-017-2612-6

**Published:** 2017-09-18

**Authors:** Amy Y. X. Yu, Hude Quan, Andrew McRae, Gabrielle O. Wagner, Michael D. Hill, Shelagh B. Coutts

**Affiliations:** 1 Department of Clinical Neurosciences, Community Health Sciences, Cumming School of Medicine, University of Calgary, Health Sciences Centre, Office 2935-B, 3300 Hospital Drive NW, Calgary, AB T2N 4N1 Canada; 2 Department of Community Health Sciences, O’Brien Institute for Public Health, Cumming School of Medicine, University of Calgary, Heritage Medical Research Building 3330 Hospital Drive NW, Calgary, AB T2N 4N1 Canada; 3 Department of Emergency Medicine, Community Health Sciences, Cumming School of Medicine, University of Calgary, Foothills Campus, 3330 Hospital Drive NW, Calgary, AB T2N 4N1 Canada; 4 Departments of Clinical Neurosciences, Community Health Sciences, Medicine, Radiology, and Hotchkiss Brain Institute, Cumming School of Medicine, University of Calgary, Health Sciences Centre, Office 2939, 3300 Hospital Drive NW, Calgary, AB T2N 4N1 Canada; 5 Department of Clinical Neurosciences, Radiology, Community Health Sciences, Hotchkiss Brain Institute, Cumming School of Medicine, University of Calgary, C1242A, Foothills Medical Centre, 1403 29th St NW, Calgary, AB T2N 2T9 Canada

**Keywords:** Transient ischemic attack, Diagnosis, Administrative data, Emergency department, Epidemiology

## Abstract

**Background:**

Validation of administrative data case definitions is key for accurate passive surveillance of disease. Transient ischemic attack (TIA) is a condition primarily managed in the emergency department. However, prior validation studies have focused on data after inpatient hospitalization. We aimed to determine the validity of the Canadian 10th International Classification of Diseases (ICD-10-CA) codes for TIA in the national ambulatory administrative database.

**Methods:**

We performed a diagnostic accuracy study of four ICD-10-CA case definition algorithms for TIA in the emergency department setting. The study population was obtained from two ongoing studies on the diagnosis of TIA and minor stroke versus stroke mimic using serum biomarkers and neuroimaging. Two reference standards were used 1) the emergency department clinical diagnosis determined by chart abstractors and 2) the 90-day final diagnosis, both obtained by stroke neurologists, to calculate the sensitivity, specificity, positive and negative predictive values (PPV and NPV) of the ICD-10-CA algorithms for TIA.

**Results:**

Among 417 patients, emergency department adjudication showed 163 (39.1%) TIA, 155 (37.2%) ischemic strokes, and 99 (23.7%) stroke mimics. The most restrictive algorithm, defined as a TIA code in the main position had the lowest sensitivity (36.8%), but highest specificity (92.5%) and PPV (76.0%). The most inclusive algorithm, defined as a TIA code in any position with and without query prefix had the highest sensitivity (63.8%), but lowest specificity (81.5%) and PPV (68.9%). Sensitivity, specificity, PPV, and NPV were overall lower when using the 90-day diagnosis as reference standard.

**Conclusions:**

Emergency department administrative data reflect diagnosis of suspected TIA with high specificity, but underestimate the burden of disease. Future studies are necessary to understand the reasons for the low to moderate sensitivity.

**Electronic supplementary material:**

The online version of this article (10.1186/s12913-017-2612-6) contains supplementary material, which is available to authorized users.

## Background

Over half of all ischemic cerebrovascular disease patients present with transient or mild symptoms [1]. Patients with transient ischemic attack (TIA) and minor stroke are at high risk of stroke recurrence or progression in the following days to weeks [2–4]. Further, despite mild or resolved symptoms, 15% of TIA and minor stroke patients develop permanent disability [5]. Accurate surveillance of TIA and minor stroke allows for monitoring disease burden and temporal trends in the population. It assists in planning health resource allocation and conducting future stroke prevention studies.

While active surveillance is defined as case ascertainment in real-time, passive surveillance is a time and cost-effective method to identify cases using existing data, such as administrative data [6]. Prior studies have shown that the coding of hospital charts using the International Classification of Diseases (ICD) has moderate to high predictive value for cerebrovascular diseases [7, 8]. However, the few validation studies that included TIA as a separate diagnostic category have found that codes for TIA in hospital discharge abstract data have lower predictive value compared to those for ischemic or hemorrhagic strokes [9–12].

Patients with TIA are increasingly being evaluated on an outpatient basis, from the emergency department (ED) to rapid-access clinics without requiring an inpatient admission [13–15]. Chart records are more sparse in the outpatient setting, which may decrease the sensitivity and specificity of passive surveillance [16, 17]. The diagnosis of TIA in the ED is challenging [18, 19] and subject to disagreement even among neurologists [20]. Although the ED is the most relevant setting to validate TIA coding, prior studies have focused on discharge diagnoses after admission to acute-care hospital and therefore are vulnerable to a specific selection bias [9, 10, 21–23].

In a cohort of patients presenting with acute and minor neurological symptoms, we aimed to determine the validity of the ICD 10th Canadian Revisions (ICD-10-CA) codes for TIA in the National Ambulatory Care Reporting System (NACRS). We hypothesized that coding for the diagnosis of TIA has lower accuracy in the ED compared to coding after inpatient hospitalization and that ED administrative data codes reflect the ED diagnosis more accurately than the 90-day diagnosis. A key secondary objective was to determine the accuracy of the ED working diagnosis compared to the final 90-day diagnosis.

## Methods

In this administrative data validation study for identifying TIA in the ED, we compared the ICD-10-CA coding algorithms to two reference standards: 1) the ED diagnosis from chart re-abstraction and 2) the final 90-day neurologist-determined diagnosis.

### National Ambulatory Care Reporting System (NACRS)

In Alberta, ED discharge diagnoses are reported in the NACRS, the Canadian national ambulatory administrative database, containing information on ED visits, day surgery admissions, and selected outpatient clinic visits [24]. The database contains one ICD-10-CA code for the “main problem” per ED visit, up to 10 codes for “other problems”, and has the option to identify queried diagnoses with a prefix “Q”. In Canada, ICD codes are assigned by non-health professional health records technicians who undergo a 2-year technical degree training. Coding is performed according to Canadian standards published and updated by the Canadian Institute for Health Information [25]. We tested four algorithms to identify TIA in NACRS: 1) TIA codes (G45.x except G45.4) in the main position without querying prefix Q (MP); 2) TIA codes in any position without Q (AP); 3) TIA codes in the main position with or without Q (MP + Q); and 4) TIA codes in any position with or without Q (AP + Q). We defined ischemic cerebrovascular disease as coding for either TIA or ischemic stroke (H34.1, H34.2, I63.x, I64.x) [10].

### Study population

The study population was obtained from two ongoing Canadian studies: SPECTRA (Spectrometry for Transient Ischemic Attack Rapid Assessment) and DOUBT (Diagnosis Of Uncertain-origin Benign Transient neurological symptoms). SPECTRA aims to identify a blood biomarker to differentiate TIA and minor strokes from mimics and enrolls patients within 24 h after symptom onset. DOUBT is a neuroimaging study of patients with TIA, minor strokes, and stroke mimics and enrolls patients within 7 days after symptom onset. Both studies prospectively enroll consenting patients and require a clinical evaluation by a neurologist at least once between symptom onset and 90 days. Study inclusion and exclusion criteria are outlined in Additional file 1: Table S1. Minor stroke is defined as National Institute of Health Stroke Scale (NIHSS) score ≤ 3. A key feature of both studies is that patients with stroke mimics are actively recruited. These are patients with acute and focal neurological symptoms in whom the working diagnosis is non-ischemic in etiology, such as syncope, seizure, migraine, or other. Recruitment for both studies largely took place in a tertiary-care teaching hospital with an ED volume of about 78,000 visits per year, including 905 annual acute stroke consults. Residents of Alberta, Canada enrolled between December 1st 2013 and October 30th 2015 with at least one ED visit prior to the enrolment date of SPECTRA (within 24 h) and DOUBT (within 7 days) were included into the current study. The Alberta unique personal healthcare number was used for deterministic linkage with NACRS. When linkage to multiple ED visits occurred, including between-ED transfers, the last visit prior to enrolment was retained for analysis. Patients who did not have a 90-day SPECTRA or DOUBT diagnosis were excluded.

### Sample size estimation

Prior studies have reported moderate to high accuracy of administrative data to identify TIA after hospital admission (77% to 97%) [9, 10]. Thus, we determined that a sensitivity or specificity below 0.85 would be a clinically meaningful cut-off. We expected a third of patients enrolled into SPECTRA and DOUBT to be true TIAs. With a sample size of 400 patients, we expected estimating sensitivity and specificity with a confidence interval width of 0.10 around the proportion estimates.

### Reference standards: ED chart re-abstraction and 90-day neurologist diagnosis

A stroke neurologist (AY) performed chart abstractions to retrospectively adjudicate an ED diagnosis, the first reference standard. The abstractor was blinded to the NACRS code and only evaluated documentation dated and timed prior to the disposition from the ED to replicate the Canadian coding standards [25]. The ED charts of four patients were missing and the SPECTRA and DOUBT case report forms were abstracted instead. The diagnosis of TIA was adjudicated based on the World Health Organization time-based criteria [26]. For example, a patient with symptoms lasting less than 24 h but a magnetic resonance imaging showing evidence of tissue ischemia, such as a small diffusion weighted imaging lesion, was adjudicated as a TIA and specifically not a stroke. TIA was further classified into “possible” or “definite,” depending on whether the adjudicator judged if there was a high likelihood of a competing non-vascular diagnosis. A senior stroke neurologist (SBC) independently reviewed a random sample of thirty charts to determine inter-abstractor reliability.

The second reference standard was the final 90-day diagnosis from the DOUBT and SPECTRA studies. In both studies, a final diagnosis was determined by a stroke neurologist at 90 days, with knowledge of all investigations and clinical follow-up completed in clinical routine.

Given the purpose of this study was to determine the validity of coding for mild or transient ischemic events, we categorized the rare intracranial hemorrhage into the stroke mimic category. These cases had a specific neuroimaging finding, heterogeneous underlying pathologies (e.g. cortical subarachnoid hemorrhage due to reversible cerebral vasoconstriction syndrome, multifocal parenchymal hemorrhage due to cerebral hyperperfusion post carotid revascularization), and all were clinically mild, NIHSS ≤3 as per inclusion criteria of the SPECTRA and DOUBT studies.

## Statistical methods

Descriptive statistics were used to determine the frequency of baseline characteristics and prevalence of disease. We first used the diagnosis from ED chart re-abstraction to determine the sensitivity, specificity, positive predictive value (PPV), and negative predictive value (NPV) of the four TIA case definition algorithms. The analyses were repeated with the final 90-day diagnosis. The categories of definite and possible TIA were combined for the primary analysis. Sensitivity analyses were performed to determine the validity of the algorithms for definite TIA as well as for ischemic cerebrovascular diseases. Accuracy of the working ED diagnosis was compared to the final 90-day diagnosis. All analyses were performed using STATA 14.0 (STATA Corp, College Station, Tex).

## Results

Among 417 patients included (*n* = 375 from SPECTRA and *n* = 42 from DOUBT), the median age was 67 years (IQR 22). Most patients, 390 (93.5%) were discharged from a tertiary comprehensive stroke centre and 27 patients were discharged from one of the four community hospitals. Re-abstracted diagnoses from ED charts showed 163 (39.1%) TIA, including 121 definite and 42 possible TIA, 155 (37.2%) ischemic strokes, and 99 (23.7%) stroke mimics. Inter-abstractor agreement was 76.7% (κ = 0.50) for the diagnosis of TIA. There were eight intracranial hemorrhages (two subarachnoid and six parenchymal hemorrhages).

Table 1 shows the baseline characteristics as well as the final 90-day diagnosis according to the adjudicated ED diagnosis. The sensitivity and specificity of the ED working diagnosis to accurately determine the 90-day final diagnosis were 61.4% [CI_95_ 52.9, 69.3] and 72.8% [CI_95_ 67.1, 78.0] for TIA; 77.3% [CI_95_ 69.1, 84.3] and 80.6% [CI_95_ 75.6, 85.0] for ischemic stroke; and 56.9% [CI_95_ 48.4, 65.2] and 93.8% [CI_95_ 90.2, 96.3] for stroke mimic.

**Table 1:** Baseline characteristics and 90-day diagnosis per ED chart adjudicated diagnosis

	TIA *n* = 163	Ischemic stroke *n* = 155	Mimic *n* = 99
Admitted to hospital n (%)	93 (57.1)	144 (92.9)	40 (40.4)
Male sex n (%)	88 (54.0)	91 (58.7)	42 (42.4)
ABCD2 ≥ 4 n (%)	119 (73.0)	134 (86.5)	54 (54.6)
Number of risk factors^a^ n (%)			
None	44 (27.0)	30 (19.4)	39 (39.4)
1	32 (19.6)	37 (23.9)	23 (23.2)
≥ 2	87 (53.4)	88 (56.8)	37 (37.4)
Neuroimage in ED n (%)	155 (95.1)	150 (96.8)	89 (89.9)
Vascular image in ED n (%)	136 (83.4)	141 (91.0)	70 (70.7)
Abnormal neuroimaging n (%)	51 (31.3)	72 (46.5)	11 (11.1)
Neurology consultation n (%)	121 (74.2)	150 (96.8)	70 (70.7)
90-day diagnosis n (%)			
TIA	89 (54.6)	42 (27.1)	14 (14.1)
Ischemic stroke	26 (16.0)	99 (63.9)	3 (3.0)
Mimic	48 (29.4)	14 (8.6)	82 (82.8)

^a^Abstracted risk factors include history of: ischemic strokes or TIA, intracerebral hemorrhage, congestive heart failure, coronary artery disease, hypertension, diabetes mellitus, dyslipidemia, atrial fibrillation, peripheral vascular disease, and active smoking. ABCD2 score: Age ≥ 60, Blood pressure, Clinical, Duration, Diabetes

Table 2 presents the sensitivity, specificity, PPV, and NPV with 95% confidence intervals of the four administrative data coding algorithms using the re-abstracted ED diagnosis. Algorithm MP identified 79 (18.9%) TIA in the cohort. It had the highest specificity (92.5%) and PPV (76.0%), but lowest sensitivity (36.8%). In comparison, algorithm AP + Q identified 151 (36.2%) TIA and had the highest sensitivity (63.8%) at the cost of lower specificity (81.5%) and PPV (68.9%). Sensitivity increased when considering all cerebral ischemia, including TIA and minor stroke, with similar specificity. The sensitivity, specificity, PPV, and NPV were overall lower when comparing administrative data with the final 90-day diagnosis as reference standard (Table 3). The results remained similar when evaluating definite TIA exclusively (Tables 4 and 5).

**Table 2:** Sensitivity, specificity, PPV, and NPV of ICD-10-CA with adjudicated ED diagnosis as reference standard

Diagnosis	Coding algorithm n (%)	Sensitivity	Specificity	PPV	NPV
TIA Prevalence 163 (39.1%)	Main position 79 (18.9%)	36.8 [29.4, 44.7]	92.5 [88.6, 95.4]	76.0 [65.0, 84.9]	69.5 [64.3, 74.4]
	Any position 99 (23.7%)	42.3 [34.6, 50.3]	88.2 [83.6, 91.9]	69.7 [59.7, 78.5]	70.4 [65.1, 75.4]
	Main position +/− Q 122 (29.3%)	53.4 [45.4, 61.2]	86.2 [81.4, 90.2]	71.3 [62.4, 79.1]	74.2 [68.9, 79.1]
	Any position +/− Q 151 (36.2%)	63.8 [55.9, 71.2]	81.5 [76.2, 86.1]	68.9 [60.8, 76.2]	77.8 [72.3, 82.7]
Cerebral ischemia Prevalence 318 (76.3%)	Main position 202 (48.4%)	60.0 [54.5, 65.5]	88.9 [81.0, 94.3]	94.6 [90.5, 97.3]	40.9 [34.3, 47.8]
	Any position 236 (56.6%)	67.3 [61.8, 72.4]	77.8 [68.3, 85.5]	90.7 [86.2, 94.1]	42.5 [35.2, 50.1]

**Table 3:** Sensitivity, specificity, PPV, and NPV of ICD-10-CA with 90-day diagnosis as reference standard

Diagnosis	Coding algorithm n (%)	Sensitivity	Specificity	PPV	NPV
TIA Prevalence 145 (34.8%)	Main position 79 (18.9%)	35.9 [28.1, 44.2]	90.1 [85.9, 93.4]	65.8 [54.3, 76.1]	72.5 [67.4, 77.2]
	Any position 99 (23.7%)	41.4 [33.3, 49.9]	85.7 [80.9, 89.6]	60.6 [50.3, 70.3]	73.3 [68.1, 78.1]
	Main position +/− Q 122 (29.3%)	49.7 [41.3, 58.1]	81.6 [76.5, 86.0]	59.0 [49.8, 67.8]	75.3 [69.9, 80.1]
	Any position +/− Q 151 (36.2%)	55.9 [47.4, 64.1]	74.3 [68.6, 79.4]	53.6 [45.4, 61.8]	75.9 [70.3, 81.0]
Cerebral ischemia Prevalence 273 (65.5%)	Main position 202 (48.4%)	63.0 [57.0, 68.7]	79.2 [71.6, 85.5]	85.2 [79.5, 89.8]	53.0 [46.1, 59.8]
	Any position 236 (56.6%)	68.9 [63.0, 74.3]	67.7 [58.3, 74.3]	79.7 [74.0, 84.6]	53.0 [45.5, 60.5]

**Table 4:** Sensitivity, specificity, PPV, and NPV of ICD-10-CA with ED definite TIA as reference standard

Diagnosis	Coding algorithm n (%)	Sensitivity	Specificity	PPV	NPV
Definite TIA Prevalence 121 (29.0%)	Main position 79 (18.9%)	45.5 [36.4, 54.8]	91.9 [88.2, 94.7]	69.6 [58.3, 79.5]	80.5 [75.8, 84.6]
	Any position 99 (23.7%)	50.4 [41.2, 59.6]	87.2 [82.8, 90.8]	61.6 [51.3, 71.2]	81.1 [76.4, 85.3]
	Main position +/− Q 122 (29.3%)	62.0 [52.7, 70.7]	84.1 [79.5, 88.1]	61.5 [52.2, 70.1]	84.4 [79.8, 88.4]
	Any position +/− Q 151 (36.2%)	67.8 [58.7, 76.0]	76.7 [71.5, 81.4]	54.3 [46.0, 62.4]	85.3 [80.5, 89.4]

**Table 5:** Sensitivity, specificity, PPV, and NPV of ICD-10-CA with 90-day definite TIA as reference standard

Diagnosis	Coding algorithm n (%)	Sensitivity	Specificity	PPV	NPV
Definite TIA Prevalence 105 (25.2%)	Main position 79 (18.9%)	41.9 [32.3, 51.9]	88.8 [84.7, 92.1]	55.7 [44.1, 66.9]	82.0 [77.4, 85.9]
	Any position 99 (23.7%)	47.6 [37.8, 57.6]	84.3 [79.8, 88.2]	50.5 [40.3, 60.7]	82.7 [78.1, 86.7]
	Main position +/− Q 122 (29.3%)	54.3 [44.3, 64.0]	79.2 [74.2, 83.5]	46.7 [37.6, 56.0]	83.7 [79.0. 87.8]
	Any position +/− Q 151 (36.2%)	60.0 [50.0, 69.4]	71.8 [66.5, 76.7]	41.7 [33.8, 50.0]	84.2 [79.3, 88.4]

The most specific algorithm MP found 19 (4.6%) false positive and 103 (24.7%) false negative cases when compared to the re-abstracted ED diagnosis. Table 6 outlines the diagnosis in the main position for the false negative cases and shows that 66% of these codes are still related to cerebrovascular diseases.

**Table 6:** First position diagnoses amongst the false negatives (*n* = 103)

Diagnoses	n (%)
Queried TIA	27 (26.2)
Stroke	26 (25.2)
Vascular abnormality^a^	15 (14.6)
Symptoms^b^	16 (15.5)
Other diagnoses^c^	19 (18.4)

^a^Intracranial or extracranial vascular occlusion, stenosis, dissection, or aneurysm

^b^One hemiplegia, 3 visual, 4 sensory, 2 gait, 2 dizziness, 1 speech, 3 malaise

^c^Ten headaches, 1 postradiation encephalopathy, 1 myelopathy, 2 vestibular dysfunction, 1 rheumatic fever, 1 aortic valve stenosis, 1 giant cell arteritis, 1 connective tissue disease, 1 seizure

## Discussions

Using the Canadian NACRS database, we showed that suspected cases of acute TIA can be identified with high specificity in the ED, but the sensitivity was low to moderate. Even the most inclusive algorithm AP + Q, evaluating codes in any position and including queried codes, only achieved a sensitivity of 64%, meaning that more than a third of suspected TIA cases discharged from the ED will be inappropriately identified as a stroke or non-stroke syndrome.

Administrative data coding can only be as good as the attending physician’s clinical diagnostic abilities. We found that administrative data reflect the ED diagnosis better than the final 90-day diagnosis, indicating that the coding reflects the episode of care. The process of translating diagnoses during the health encounter into ICD codes is a static snapshot in time, while a physician’s diagnostic impression is fluid and changes based on new information from the clinical course, collateral history, and investigation results. Empirically, transient neurological events are described and diagnosed by the clinical history and show high diagnostic variability. As a result, we show an ED diagnosis of TIA carries only a 61% sensitivity and 73% specificity compared to the final diagnosis, a finding that is consistent with prior reports [18–20].

Our results have implications for the use of administrative data for TIA surveillance and research. Suspected acute TIAs can be identified with high specificity and used to determine the temporal trends of disease, but the low sensitivity will underestimate the burden of disease. The most inclusive algorithm AP + Q has moderate sensitivity 64% and PPV 69% and may be useful to evaluate patterns of healthcare utilization in suspected acute TIA or to determine adherence rates to secondary stroke prevention guidelines. However, because the coding is less accurate when compared to the 90-day diagnosis, if administrative data were used to select a cohort of suspected TIAs for a prospective study to determine outcomes of interest, such as rates of stroke recurrence or mortality after TIA, misclassification errors must be addressed by chart review or other case ascertainment method. Finally, there are implications for the use of administrative data for other conditions primarily managed in the ED. Outpatient cases, for example angina or a single seizure, should be validated separately from their inpatient counterparts, acute myocardial infarction or complex seizure disorder [27, 28].

We show that all four algorithms yielded a similar PPV (68.9 to 76.0%), which is lower than the estimates reported by prior TIA validation studies on discharge diagnostic codes after hospital admission (88.9 to 97%) [9, 10], but higher than other studies on TIA patients discharged from the ED (48.8 to 56.1%) [12, 29]. Our study used different methods compared to prior publications. By including patients with TIA mimics, we were able to determine both true negative and false positive cases, allowing for the calculation of sensitivity and specificity. The majority of prior studies evaluating the validity of administrative data in cerebrovascular diseases identified a priori patients with high-yield stroke codes and then verified the coding accuracy or agreement [9, 10, 23, 30, 31]. By design, these studies had a high prevalence of disease, which may lead to an overestimation of the agreement [32] and PPV [33]. Others examined the validity of cerebrovascular disease coding as a comorbidity of another condition (e.g. diabetes, hypertension), such that the prevalence of cerebrovascular diseases is low, less than 5%, yielding estimates with wide confidence intervals and prevalent disease is not differentiated from incident cases [34, 35]. Finally, a third type of study validated administrative data against a registry or other active case finding methods [11, 12, 16, 36]. Stroke mimics are excluded by design, limiting the ability to evaluate true negative and false positive cases.

Our study has several strengths. By using two reference standards, we addressed the question of accuracy of administrative data in two different ways: how well do the codes reflect 1) the clinical encounter and 2) the patient’s actual condition? By including stroke mimics, we evaluated true negative and false positive rates in order to report specificity estimates. Some limitations are worth mentioning. SPECTRA and DOUBT prospectively enrolled patients who consented to participation, resulting in a sample of non-consecutive patients presenting to the ED with acute neurological symptoms. The TIA prevalence in our study is higher than that of a randomly selected sample from the ED; because prevalence impacts the predictive value, our results may not be directly applicable to a specific ED population. Nevertheless, the prevalence of TIA and stroke mimics in this study is similar to prior publications on patients with acute neurological presentations referred to a stroke prevention clinic [19, 37]. The majority of the patients were discharged from a single center. This may limit the generalizability of our results because administrative data coding is variable between jurisdictions. Finally, although chart abstractors were blinded to NACRS codes and the 90-day diagnosis, the retrospective derivation of the ED diagnosis may be subject to residual bias.

## Conclusions

We show that administrative data identify suspected TIA in the ED with high specificity while underestimating the incidence of acute TIA cases. We also highlight the diagnostic challenges that are presented to the physician for patients with TIA in the ED. Understanding the detailed reasons for the moderate sensitivity of administrative data for this diagnosis and whether the miscoded patients are systematically different from the accurately coded ones will require prospective diagnostic studies.

## Additional file

Additional file 1: Table S1. Inclusion and exclusion criteria for the DOUBT and SPECTRA studies. Table listing the inclusion and exclusion criteria for the DOUBT and SPECTRA studies. (DOCX 22 kb)

## Abbreviations

DOUBT: Diagnosis Of Uncertain-origin Benign Transient neurological symptoms
ED: Emergency Department
ICD: International Classification of Diseases
ICD-10-CA: International Classification of Diseases, 10th Canadian iteration
NACRS: National Ambulatory Care Reporting System
NPV: Negative Predictive Value
PPV: Positive Predictive Value
SPECTRA: Spectrometry for Transient Ischemic Attack Rapid Assessment)
TIA: Transient Ischemic Attack
