# Supplementary material for: Moderate sensitivity and high specificity of emergency department administrative data for transient ischemic attacks
Source: BMC Health Serv Res. 2017 Sep 18;17:666. doi: 10.1186/s12913-017-2612-6 (PMC5604304; doi:10.1186/s12913-017-2612-6)
Supplement: Additional file 1: Table S1. — Inclusion and exclusion criteria for the DOUBT and SPECTRA studies. Table listing the inclusion and exclusion criteria for the DOUBT and SPECTRA studies. (DOCX 22 kb) [file 12913_2017_2612_MOESM1_ESM.docx]

Table S1 Inclusion and exclusion criteria for the DOUBT and SPECTRA studies

|  | **DOUBT** | **SPECTRA** |
| --- | --- | --- |
| **Inclusion criteria** | - Transient focal neurological deficits: Including at least one of focal sensory symptoms, visual symptoms, diplopia, vertigo, limb clumsiness (without weakness), gait instability of any duration, or weakness (clear motor weakness) or speech disturbance (aphasia or dysarthria) only if lasting less than or equal to 5 minutes **OR** - Ongoing focal neurological symptoms at the time of referral that do not include weakness, aphasia or dysarthria. Symptoms must include at least one of focal sensory symptoms, visual symptom, diplopia, vertigo, limb clumsiness (without weakness) or gait instability of any duration. + NIHSS 0-3 **AND** - The evaluating neurologist estimates that a vascular event (TIA or minor stroke) is a possibility in the differential diagnosis (not necessarily a likely diagnosis) - Patient interviewed and examined by a neurologist within 7 days - 40 years old or older - Informed consent | Participants will be eligible to participate if all of the following conditions are present:   - Age 18 and older - Suspected TIA or mild stroke (NIHSS≤3) - English speaking or translator available - Competent to provide consent and report symptoms - Be able to provide blood sample for the study within 24 hours after symptom onset |
| **Exclusion criteria** | - Motor weakness or speech symptoms lasting more than 5 minutes involved in the event - Isolated visual loss that is clearly monocular (patient can be enrolled if possibly a hemianopia or if another focal neurological symptom occurred with it) - Prior clinical stroke (old asymptomatic stroke on imaging is not an exclusion criteria) - Modified Rankin scale 2 or more. The patient must be fully independent of activities of daily living at the time of event, must not live in a nursing home and be cognitively intact - Serious co-morbid illness where the participant would be unexpected to survive 12 months and complete follow-up (eg. Metastatic cancer) - The examining neurologist concludes that the diagnostic criteria are met for an alternative etiology (carpal tunnel, BPPV with positive Dix Hallpike, radiculopathy etc.) - Patient is unable or unwilling to undergo MRI examination within 7 days of symptom onset. Patient has a contraindication to MRI examination (eg. Pacemaker or ICD, significant renal failure making contrast MRI unsafe) | Participant will be excluded from participating in the study if any of the following conditions are present:   - Stroke severity exceeding 4 on the NIHSS scale - Unable to obtain MRI (within 7 days) or CT/CTA (within 24 hrs) - Non-English speaking, unless translator present - Isolated monocular blindness |
